# Congress Internationale De La Tuberculosis, France

**Published:** 1904-03

**Authors:** 


### Congress Internationale De La Tuberculosis, France.

We are glad to be able to announce that this very powerful body, which was announced to meet in Paris, on September 26 to October 1, 1904, under the presidency of Professor Brouardel, one of the honorary members of the Medico-Legal Society, and of whom Prof. Dr. M. Letulle, Professor Aggerge, of the faculty of medicine 7 Rue de Magdebourg is secretary, has decided to postpone its session to the year 1905. Professor Brouardel has been advised of the action of the United States government respecting the holding of the American Congress at the St. Louis Exposition, at dates, which, while although not identical, were too near each other to enable the Frenchmen to attend the St. Louis Congress or the Americans to attend the Paris Congress.

The adjournment will accommodate both bodies and enable the International Congress at St. Louis to receive the representatives of the Paris Congress, as it will be invited to be represented.

There has been for many years a great desire to meet Professor Brouardel on this side of the Atlantic, and it is to be hoped that he will attend the International Congress at St. Louis, on October 3, 4 and 5, 1904.—*Medico-Legal Journal, December, 1903.*

